# From Inflammation to Fibrosis—Molecular and Cellular Mechanisms of Myocardial Tissue Remodelling and Perspectives on Differential Treatment Opportunities

**DOI:** 10.1007/s11897-017-0343-y

**Published:** 2017-07-13

**Authors:** Navin Suthahar, Wouter C. Meijers, Herman H.W. Silljé, Rudolf A. de Boer

**Affiliations:** Department of Cardiology, University Medical Center Groningen, University of Groningen, PO Box 30.001, 9700 RB Groningen, The Netherlands

**Keywords:** Cardiac, Fibrosis, Inflammation, Macrophages, HF, ECM, Heart

## Abstract

**Purpose of Review:**

In this review, we highlight the most important cellular and molecular mechanisms that contribute to cardiac inflammation and fibrosis. We also discuss the interplay between inflammation and fibrosis in various precursors of heart failure (HF) and how such mechanisms can contribute to myocardial tissue remodelling and development of HF.

**Recent Findings:**

Recently, many research articles attempt to elucidate different aspects of the interplay between inflammation and fibrosis. Cardiac inflammation and fibrosis are major pathophysiological mechanisms operating in the failing heart, regardless of HF aetiology. Currently, novel therapeutic options are available or are being developed to treat HF and these are discussed in this review.

**Summary:**

A progressive disease needs an aggressive management; however, existing therapies against HF are insufficient. There is a dynamic interplay between inflammation and fibrosis in various precursors of HF such as myocardial infarction (MI), myocarditis and hypertension, and also in HF itself. There is an urgent need to identify novel therapeutic targets and develop advanced therapeutic strategies to combat the syndrome of HF. Understanding and describing the elements of the inflammatory and fibrotic pathways are essential, and specific drugs that target these pathways need to be evaluated.

## Introduction

Heart failure (HF) is a leading cause of morbidity and mortality worldwide and an important cause of hospitalization. It severely reduces the quality of life of the affected and the 5-year mortality rate is higher than that of most malignancies [1–3]. Various types of cardiac insults culminate in the syndrome of HF, but inflammation and fibrosis are key pathophysiological mechanisms operating in the failing heart. These mechanisms affect the tissue architecture, electrical conduction and mechano-electrical coupling and also have direct deleterious effects on force generation by cardiomyocytes [4].

In this review, we focus on important cellular and molecular mechanisms of cardiac inflammation and fibrosis, the interplay between inflammation and fibrosis in various precursors of HF such as myocardial infarction (MI), hypertension and myocarditis and how persistence of such mechanisms could enhance progression to chronic HF (CHF). Furthermore, we provide insights into novel therapeutic options currently available and those being developed to combat HF.

## Inflammation and Fibrosis

### Inflammation

Inflammation is a physiological defence mechanism of the body against injurious stimuli such as tissue damage and infection. Timely inflammation in adequate intensity is essential to eliminate harmful stimuli; an insufficient inflammatory response can result in persistence of the trigger. Active resolution of inflammation is also essential as it facilitates tissue healing after injury; failure to resolve leads to chronic inflammation, extended tissue destruction and progressive fibrosis [5, 6]. Inflammation and fibrosis can thus be viewed as a continuum of events within the framework of tissue defence, repair and regeneration.

The inflammatory response is extremely complex and comprises several stages including vascular phase, cellular phase and resolution phase. Leukocytes are major cellular effectors that direct this response through various mechanisms, including chemical mediators such as cytokines [7]. During the inflammatory process, the endothelial layer undergoing activation and selective changes in permeability allows cellular components to shift from intravascular to extravascular compartment [8]. Secreted proteins and extracellular matrix (ECM) components also play a vital role in inflammation by directly moderating the inflammatory cascade or by providing signals to cellular components of inflammation. Osteopontin, a phosphorylated glycoprotein secreted by monocytes and lymphocytes, mediates leukocyte adherence and migration [9]. Versican is an ECM proteoglycan, also involved in leukocyte adherence and migration; it is abundantly expressed and produced by activated macrophages and stromal cells during inflammation [10]. Hyaluronic acid (HA) is a glycosaminoglycan ECM component having a dual role in inflammation. While native polymeric HA is typically anti-inflammatory [11, 12], the smaller fragments elicit a pro-inflammatory response by binding to toll-like receptor 2 (TLR2) and TLR4 of monocytes, dendritic cells and lymphocytes [13]; TLRs are a class of proteins that play a key role in the innate immune system. Recent studies also indicate that low molecular weight HA fragments promote a classically activated “pro-inflammatory” state in macrophages [14].

### Resolution of Inflammation

**Fig. 1:**
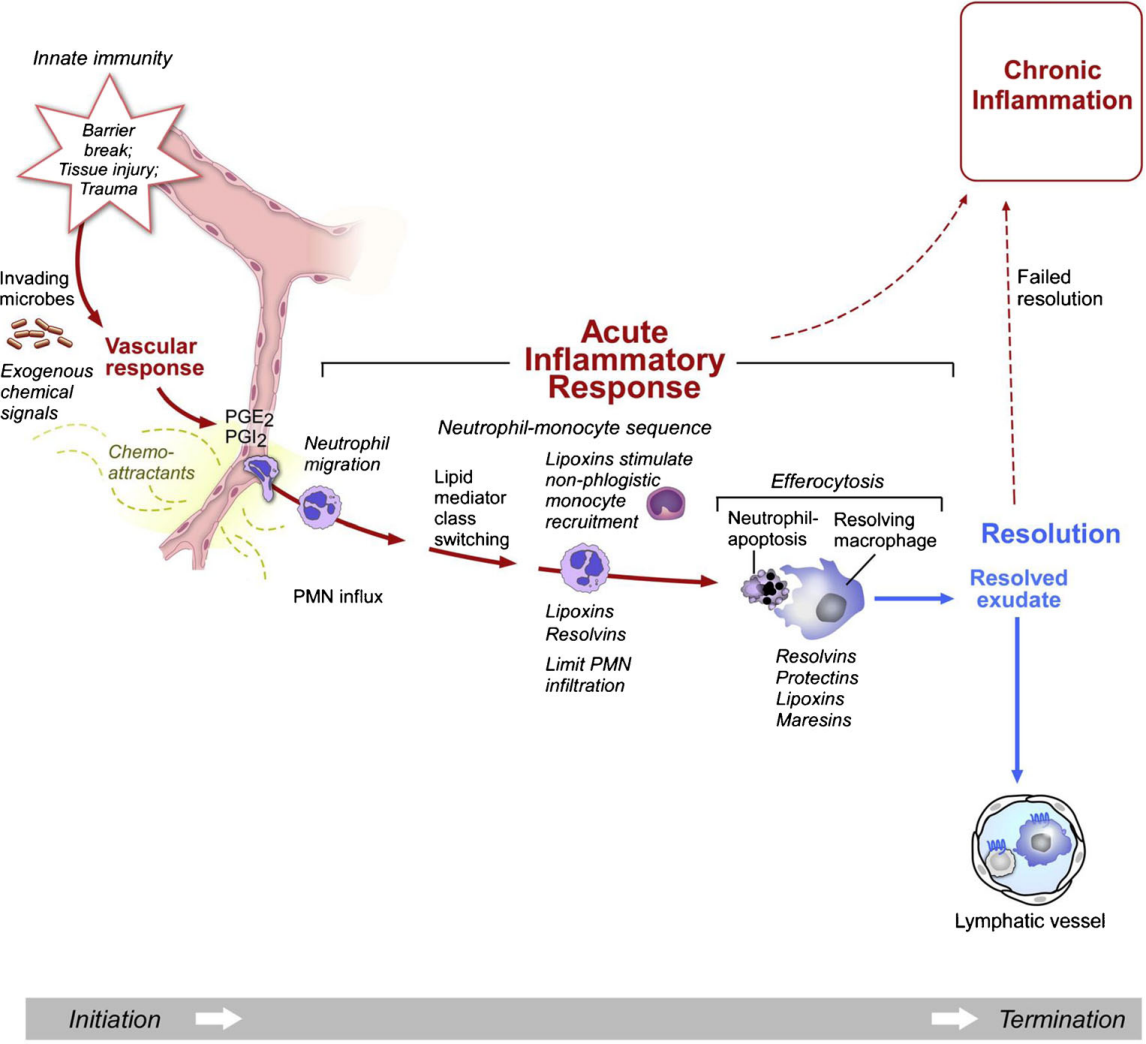
A simplified depiction of sequence of events in an inflammatory response and the role of proresolution mediators in its termination. Tissue injury elicits an initial vascular response, followed by an influx of neutrophils and monocytes to the damaged area. After reaching the tissue, monocytes transform into macrophages and actively phagocytose the debris. Lymphocytes, which are cells of the adaptive immune system, later modulate this initial response. The basic mechanisms of resolution of inflammation are highlighted which are (1) lipid mediator class switching producing proresolution molecules such as lipoxins and resolvins; (2) increased efferocytosis by macrophages; (3) anti-inflammatory cytokines secreted by “resolving” macrophages and regulatory T cells. Failure to resolve leads to persistence of inflammation resulting in a chronic inflammatory state, causing sustained tissue injury. Adapted figure reproduced from [17] Buckley et al. 2014 with permission from the authors. PGE2, prostaglandin E2; PGI2, prostacyclin

Resolution of inflammation is an active process orchestrated by “pro-resolution” factors. These factors induce “pro-resolution” programmes in stromal cells and provide cues to inflammatory cells such as neutrophils to undergo apoptosis. They also enhance efferocytosis and later signal macrophages to exit via lymphatic vessels [6, 15••]. Polyunsaturated fatty acid-derived resolvins and protectins function as proresolution factors and play a key role in subduing inflammation [16, 17] (Fig. 1). Inflammation is further modulated by a number of checkpoints. For instance, TLR-mediated inflammasome activation is countered by a negative internal feedback mechanism involving phosphoinositide 3-kinase (PI3K) and excessive TLR signalling is moderated by negative regulators of immune responses, such as interleukin-1 receptor-associated kinase-M (IRAK-M) and suppressor of cytokine signalling-1 (SOCS-1). T-regulatory cells also actively inhibit inflammation by producing several anti-inflammatory cytokines [18, 19]. Failure of such regulatory mechanisms could lead to a state of chronic inflammation causing continuous tissue damage and progressive fibrosis.

### Fibrosis

Fibrosis is an essential component of tissue repair that follows tissue injury and is usually associated with inflammation. The aim of fibrosis is to deposit connective tissue in order to preserve tissue architecture; progressive fibrosis reflects a pathologic state and results in scarring, impairment of function and organ damage [5, 20].

Myofibroblasts are major cells responsible for ECM secretion; they arise directly from fibroblasts or from other cell types such as macrophages, endothelial cells, pericytes and circulating monocytes. Several literature reviews exclusively discuss the role of (myo)fibroblasts in fibrosis and interested readers are directed to them [21, 22•].

Macrophages also play a pivotal role in secretion of ECM components and in ECM remodelling. They are major sources of matrix metalloproteinases (MMPs) and tissue inhibitor of metalloproteinases (TIMPs) [23] and are the primary cells involved in the phagocytosis of cellular debris and infectious agents. The phagocytosed particle can influence phenotypic characteristics of macrophages [24, 25]; for instance, macrophages assume a more fibrotic (M2) phenotype after ingesting apoptotic neutrophils [26]. Cytokines such as interleukin-13 (IL13) and IL4 also induce profibrotic (M2) phenotypic changes in naïve (M0) macrophages. M2 phenotype is characterized by reduced expression and secretion of inflammatory mediators, e.g. tumour necrosis factor-α (TNFα) and IL6, and augmentation of cell survival and fibrotic signals, e.g. IL10, insulin-like growth factor-1 (IGF1), transforming growth factor-β (TGFβ) and galectin-3 (Gal-3) [27•, 28•]. Besides promoting fibrosis, M2 macrophages also endocytose collagen utilizing mannose receptors highlighting their pleiotropic role in ECM homeostasis [29]. Other immune cells, e.g. neutrophils, lymphocytes and eosinophils, also contribute to the development of fibrosis in various organs [7]. Extensive communication between inflammatory cells, fibroblasts and ECM actively modulates the fibrotic response [30–33].

## Cardiac Inflammation

Virtually any cardiac insult, e.g. ischaemia and infection, can initiate an inflammatory response in the heart; systemic inflammation can in itself trigger several inflammatory pathways within the cardiac tissue [34]. While acute cardiac inflammation, e.g. myocarditis, could result in rapid decline of cardiac function, chronic inflammation causes progressive structural damage, leading to cardiac fibrosis.

### The Role of Various Cell Types in Cardiac Inflammation

a. Immune cells as a source of cardiac inflammation: Neutrophils and monocytes home to the site of cardiac injury and release aggressive mediators such as reactive oxygen species (ROS) and proteases, with the primary aim of eliminating the factors that caused the cardiac insult. However, this nonspecific response could also result in extensive damage to the healthy cardiac tissue [35]. Macrophages exposed to inflammatory signals, e.g. Interferon-γ (IFNγ) and IL1, typically assume a pro-inflammatory M1 phenotype [36]. These macrophages sustain cardiac inflammation by secreting inflammatory cytokines themselves and can also signal neighbouring fibroblasts and cardiomyocytes to adopt pro-inflammatory phenotypes [37]. In subsequent stages of inflammation, effectors of innate immune system are modulated by lymphocytes; for instance, cytokines secreted from Th1 cells sustain inflammation while Th2 cytokines produce anti-inflammatory and prohealing signals [38].

b. Pro-inflammatory cardiomyocytes in cardiac injury: Cardiomyocytes (~30–40% of cells in the healthy heart) secrete pro-inflammatory cytokines typically after hypoxia or cardiac injury [39•]. TNFα expression is upregulated in hypoxic cardiomyocytes [40] while lipopolysaccharide (LPS) stimulation of cardiomyocytes in vitro increases IL6 production [39•]. IL6 and other related cytokines secreted by cardiomyocytes are pivotal in regulating cardiac myocyte hypertrophy and apoptosis [41]. Moreover, IL6 is known to direct the nature of inflammation from acute to chronic, by changing the leukocyte infiltrate from neutrophils to monocyte/macrophages [42].

c. Cardiac fibroblasts as a source of pro-inflammatory cytokines: Although mentioned frequently in the context of fibrosis, cardiac fibroblasts exposed to an inflammatory milieu, e.g. TNFα, transform to a pro-inflammatory phenotype, with increased expression of cytokines such as IL1β and IL6 [43]. When activated by mechanical stress, they produce pro-inflammatory mediators such as monocyte chemoattractant protein-1 (MCP-1), IL8 and biglycan [44••]. Cardiac fibroblasts also sustain and perpetuate pre-existing inflammation; they directly facilitate transendothelial migration of leukocytes by producing gelatinases such as MMP9. Co-culture of fibroblasts with macrophages also increases macrophage inflammatory protein-1α (MIP-1α) expression in macrophages and enhances reciprocal enhancement of monocyte-fibroblast adhesion and chemokine production [30]. Furthermore, cardiac fibroblasts stimulated by IL-17A produce chemokines such as MCP-1, IL6 and leukaemia inhibitory factor (LIF), responsible for recruiting and differentiating myeloid cells, and this mechanism has been implicated in the pathophysiology of inflammatory dilated cardiomyopathy (IDCM) [45].

### Cardiac Inflammatory Pathways

TLRs are a part of the innate immune system and play a crucial role in the development of inflammatory disorders by initiating both innate and adaptive immune responses. They are essentially pattern recognition receptors (PRRs) designated to recognize infectious or dangerous foreign patterns collectively termed as pathogen-associated molecular patterns (PAMPs) and danger-associated molecular patterns (DAMPs) [46]. TLR4 is usually expressed in monocyte-macrophage-lineage cells also in fibroblasts and epithelial cells. Recent work by Liu and colleagues demonstrate upregulation of TLR4 in cardiomyocytes in HF [47••]. Lipid A component is an important exogenous ligand for TLR4, while various intracellular and extracellular components (e.g. heat shock proteins (HSP), fibrinogen, heparin sulphate, HA) serve as endogenous ligands [48]. Intracellular TLR4 signalling can occur via both the *myeloid differentiation primary response gene-88* (MyD88)-dependent pathway resulting in early *nuclear factor-κβ* (NFκβ) activation or the MyD88-independent pathway resulting in late NFκβ activation [49].

TNF-NFκβ pathways are indicated in cardiac infection and injury, while viral triggers typically activate retinoic acid-inducible gene-1 (RIG-1) pathways. Other cardiac inflammatory mechanisms include caspase-1-inflammasome pathways, activated usually during oxidative and cellular stress [50]. Persistent activation of various cardiac inflammatory pathways could serve as a precursor to fibrotic changes, resulting in pathological remodelling of the heart.

## Cardiac Fibrosis

Myocardial fibrosis can be classified as reactive interstitial fibrosis, replacement fibrosis and perivascular fibrosis [22•]. Extensive cardiac fibrosis results in electro-mechanical disturbances and reduces nutrient supply toward the myocardium, perpetuating a vicious cycle of fibrosis, cell death and inflammation [51]. Herein, we briefly discuss the role of cardiac fibroblasts, macrophages, angiogenesis and matricellular components in cardiac fibrosis.

a. Cardiac myofibroblasts: Fibroblasts comprise up to 60–70% of the cellular population in the heart. Activation of cardiac fibroblasts to α-smooth muscle actin (α-SMA) expressing myofibroblasts is a crucial step toward fibrosis [39•]. Collagen-producing myofibroblasts typically develop after cardiac injury and are programmed to undergo apoptosis after carrying out their reparative “tissue-building” activities. Persistence of myofibroblasts leads to progressive fibrosis [52].

Sustained activation by mechanical stress or by profibrotic molecules from neighbouring myofibroblasts and macrophages (e.g. TGFβ, Gal-3) results in transformation of quiescent fibroblasts into active collagen-producing myofibroblasts [27•, 28•]. A recent study by Tian et al. revealed that sirtuin-6 (SIRT6) depletion in cardiac fibroblasts by SIRT6 siRNA increased the expression of α-SMA, resulting in a myofibroblast phenotype [53]. Extensive work done by Herum and colleagues demonstrate for the first time, the involvement of syndecan-4 in cardiac fibroblast-myofibroblast conversion upon mechanical stress [54••]. Profibrotic properties of cardiac fibroblasts are also potentiated by syndecans. Overexpression of syndecan-4 in cardiac fibroblasts induces overexpression of collagen, osteopontin and lysyl oxidase (LOX) and is deemed to be a key player in the development of passive myocardial stiffness in the pressure-overloaded heart [54••].

Crosstalk between fibroblasts and cardiomyocytes is also important in cardiac remodelling; myofibroblasts induce and modify cardiomyocyte hypertrophy through such mechanisms [55, 56]. Cardiac fibroblast-cardiomyocyte crosstalk occurs via biochemical interactions involving paracrine factors such as TGFβ, angiotensin-II (Ang II) and interleukins. Fibroblast-cardiomyocyte signal transduction also occurs via electro-mechanical interactions utilizing gap junction proteins such as connexins 43 and 45 or through biomechanical interactions [22•, 57].

b. Cardiac macrophages and cardiac mast cells: Macrophages are heterogenous and are phenotypically and functionally diverse, and the M2 macrophage phenotype is closely associated with fibrosis. Utilizing a mouse model of hypertension, Falkenham and colleagues demonstrated that M2 resident cardiac macrophages play a pivotal role in the development of myocardial fibrosis [58]. Moriwaki et al. utilized transgenic ApoE^−/−^ mice that overexpressed urokinase-type plasminogen activator (uPA) in macrophages. In comparison to that of controls, their hearts were bigger and had a significant amount of macrophage infiltration and increased collagen content. This effect was cardiac specific, as other organs of transgenic mice did not display a higher amount of inflammation and fibrosis in comparison to those of controls. Plasminogen activator inhibitor-1 (PAI-1)-deficient mice also developed exclusive fibrosis of the heart; fibrosis was absent in the liver, spleen, lungs and kidneys. This suggests that balance between uPA and its inhibitor PAI-1 is important in homing of macrophages to the cardiac tissue and for the development of cardiac fibrosis [59••]. Carlson et al. recently demonstrated that in infarcted mice and human hearts, there is a direct association between cardiac M2 macrophages and fibrosis [60]. In this context, it is interesting to note that macrophages do not usually undergo apoptosis and exit via lymphatic vessels. Thus, a well-functioning cardiac lymphatic drainage is also of importance to curb fibrosis associated with chronic inflammation [61].

Although a lot is not known about cardiac mast cells, they appear to have a dual role in cardiac fibrosis. They tend to be antifibrotic in the healthy heart and promote fibrosis in the injured or diseased cardiac tissue [62, 63].

c. Role of angiogenesis: Impaired angiogenesis and insufficient neovascularization result in inadequate delivery of oxygen and nutrients to the failing heart. Cardiomyocyte loss follows, and a vicious cycle of oxidative stress, cell death and fibrosis ensues [64]. In a rat model of HF after MI, treatment with erythropoietin improved cardiac function by inducing neovascularization [65]; in patients with acute MI, high serum erythropoietin levels were associated with a smaller infarct size [66]. Several therapeutic strategies that improve angiogenesis are currently being developed to treat cardiac fibrosis and HF [67, 68].

d. Matricellular components: The myocardial matrix is very complex and dynamic. Myocardial matricellular proteins, together with various regulatory proteins, are indicated in the development or attenuation of cardiac fibrosis [69]. For example, thrombospondins (TSP) are matrix glycoproteins involved in cardiac remodelling occurring after cardiac stress or injury. TSP1 is known to convert latent TGFβ to its active form and is indicated extensively in cardiac remodelling [32, 70]. Frolova et al. demonstrated the important role of TSP4 in reactive fibrosis caused by pressure overload to the heart in a transverse aortic constriction (TAC) mouse model [71]. Other matricellular components such as osteopontin and periostin are also profibrotic and remain elevated in pathophysiological scenarios such as MI and HF [31, 72]. Biglycan and decorin are closely related ECM proteins belonging to the family of small leucine-rich proteoglycans (SLRP) yet having different properties with respect to cardiac remodelling and fibrosis. Although biglycan is an indispensable player in adaptive remodelling after MI [73], ablation of this protein in the setting of left ventricular pressure overload attenuates cardiac hypertrophy [74]. Extracellular decorin, however, has an antifibrotic effect and inhibits the action of TGFβ on human cardiac fibroblasts. Decorin also “reverses” adverse cardiac remodelling in the failing human heart, highlighting its role in antagonizing cardiac fibrosis [75•].

ECM-cellular interactions are tightly regulated by modulatory proteins such as Gal-3 and syndecans. Gal-3 is a matricellular glycan-binding protein involved in cardiac fibrosis and remodelling [76, 77••]. Activation of Gal-3 results in its multimerization and formation of Gal-3 lattices on cellular surfaces. Apart from critically regulating exchange of information between cellular and extracellular compartments, Gal-3 lattice can also amplify fibrotic signalling. A suggested mechanism is lattice entrapment of TGFβ receptors, resulting in amplification of profibrotic signalling pathways [33, 78]. Recent studies also indicate extensive interactions between Gal-3 and various other ECM components such as sulphated glycosaminoglycans and chondroitin sulphate, indicating Gal-3 as a glycosaminoglycan-binding protein (GAGBP) [79]. However, further studies are needed to clarify if such interactions also modulate ECM remodelling. Syndecans are cell-associated transmembrane proteoglycans that are usually involved in cell-matrix interactions. Syndecan-4 and syndecan-1 are indicated extensively in cardiac fibrosis [80, 81]. Syndecan-1 amplifies Ang II–TGFβ signalling in angiotensin II-mediated cardiac fibrosis via an unknown mechanism [82] while syndecan-4 increases collagen cross-linking leading to passive myocardial stiffness [54••].

**Fig. 2:**
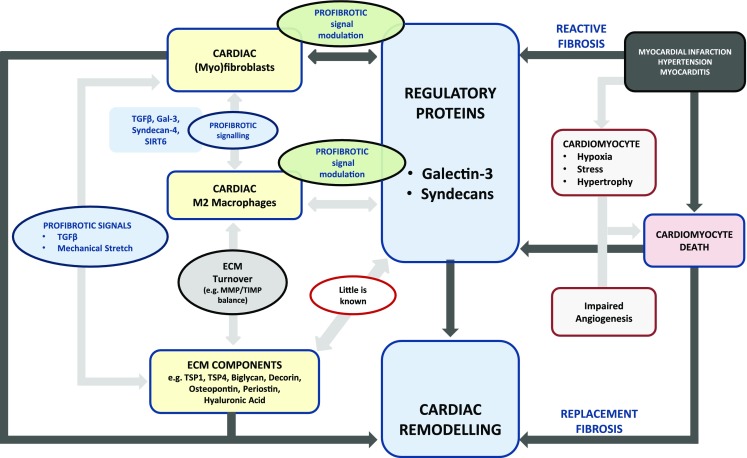
Basic mechanisms of cardiac fibrosis highlighting the role of regulatory proteins in profibrotic signal modulation. ECM matrix deposition is the hallmark of fibrosis and myofibroblasts are the central cells in ECM synthesis. M2 macrophages also play a crucial role in fibrosis and influence ECM turnover chiefly by influencing MMP/TIMP proportions. There is extensive communication between these two cell types occurring through direct cell-cell interactions and also through paracrine signalling. In this diagram, we emphasize the central role of regulatory proteins, such as galectin-3 and syndecans, and how they can directly moderate fibrotic signalling between myofibroblasts and M2 macrophages. However, little is known about the interaction of regulatory proteins directly with ECM components and this could be the focus of future research. Two commonly occurring fibrotic scenarios in the heart are also depicted. In reactive fibrosis, cardiomyocyte death is usually the consequence of fibrosis; while in replacement fibrosis, cardiomyocyte death is the key driver of fibrosis. ECM, extracellular matrix

Thus, it appears that ECM components together with modulatory proteins play a crucial role in the development and resolution of the profibrotic response in the heart. Although a substantial amount of information is known about ECM signalling in fibrosis, there are still several missing links and avenues for exploration (Fig. 2).

## From Inflammation to Fibrosis in Major Scenarios of Cardiac Injury

The sequel from inflammation to fibrosis in various cardiac disease scenarios is different depending on the nature of cardiac insult and its duration. A deeper understanding of the mechanisms and succession of events could help us identify possible therapeutic targets and increase treatment possibilities. Herein, we discuss dominant scenarios of cardiovascular injury, namely MI, myocarditis and hypertension, and how persistence of inflammation could lead to progressive fibrosis and HF.

### Myocardial Infarction

MI usually occurs after a vascular insult to the myocardium and is characterized by extensive necrosis of cardiomyocytes. This results in leakage of intracellular contents and accumulation of ROS. The released DAMPs together with cytokine signals from the neighbouring cells constitute the “alarmin-response” [83]. However, the infarcted area has limited or no vascularization, and this prevents the blood-borne immune cells from gaining immediate access to the necrotic core. During these initial stages of ischaemic damage, cardiac myofibroblasts could potentially take over the phagocytic role of macrophages, by actively engulfing dead cells [84].

This is followed by an intense and transient inflammatory phase, characterized by a “neutrophil-monocyte” infiltration [85]. However, both resident cardiac cells (cardiomyocytes, cardiac fibroblasts, resident macrophages, mast cells) and recruited cells (leukocytes) contribute to the development of sterile inflammation post-MI [85, 86•]. The innate immune cells recognize the released alarmins utilizing TLRs and activate downstream inflammatory pathways, and TLR2 and TLR4 are crucial players in the post-infarct inflammatory reaction.

Inflammation is further sustained by upregulation of various pro-inflammatory cytokines, e.g. MCP-1, TNFα and IL6, within the infarcted myocardium. MCP-1 is involved in the recruitment of monocytes while TNFα enhances adhesion and extravasation of leukocytes through the endothelium [87–89]. TNFα is an acute-phase protein involved in both post-MI inflammatory reaction and ischaemia-reperfusion (I/R) injury [90, 91•]. The role of IL6 in cardiac inflammation and remodelling is ambiguous. Enhanced IL6 expression could accentuate the inflammatory response and exacerbate the deleterious after-effects of MI [42, 56]. However, knocking out IL6 confers no protective effect in a mouse model of MI [92]. Moreover, IL-6 receptor inhibition did not improve cardiac function after I/R in a recent study [93]. There are also changes in ECM around the necrotic area after MI. For instance, large polymers of HA are degraded to low molecular weight HA and together with fibronectin fragments initiate and sustain a multitude of inflammatory cascades [94].

Molecular stop signals of inflammation such as IRAK-M in macrophages and fibroblasts actively wean the post-MI inflammatory response. They prevent uncontrolled TLR/IL1-mediated responses by acting as a functional decoy to attenuate sustained inflammatory response and improve adverse post-infarction cardiac remodelling [95].

In the proliferative phase that follows, macrophages secrete several cytokine growth factors and activate mesenchymal reparative cells to deposit ECM [85]; Gal-3, a profibrotic protein produced predominantly by macrophages, is a major player in post-MI cardiac remodelling [27•, 77••, 96]. TGFβ, another key fibrotic cytokine, aids in repair by supressing inflammation and stimulating hypertrophic cardiomyocyte growth after MI. TGFβ also promotes ECM deposition by upregulating collagen and fibronectin synthesis and downregulating ECM degradation [28•, 97]. Crosstalk between M2 macrophages and fibroblasts together with Th2 responses sustains the fibrotic response. Recent studies also suggest the indispensable role of proteoglycans such as syndecan-1 and 4 in post-MI remodelling and fibrosis of the heart. Although mice lacking syndecan-1 and 4 showed marked reduction in profibrotic signalling, this resulted in increased cardiac rupture after MI [80, 81].

Apoptosis of the majority of reparative cells marks the end of the proliferative stage and infarct maturation occurs with the formation of cross-linked collagen. The extent of post-MI remodelling depends on the infarct size and the quality of cardiac repair. The infarct zone undergoes replacement fibrosis while the surrounding non-infarct zone displays perivascular and interstitial fibrosis [98•]. The aim of the fibrotic response is to preserve structural integrity and to maintain the pump function of the heart by preventing dilatation, aneurysm formation or myocardial rupture [99]. However, failure of cardiac myofibroblasts to undergo apoptosis or persistence of profibrotic signalling could result in pathological remodelling of the heart.

### Myocarditis—Inflammatory Cardiomyopathy

Viral infection is a common cause of myocarditis and is characterized by inflammation of the myocardium; we discuss the sequence of events from infection to fibrosis in group B Coxsackie viral (CVB) infection.

Macrophages and lymphocytes of Peyer’s patches and the spleen serve as ports of entry for CVB3 viral particles, and they reach the heart through the bloodstream. Utilizing endothelial receptor CAR (coxsackievirus and adenovirus receptor), primarily located in the intercalated discs of the adult heart or receptor DAF (delay accelerating factor), they translocate into cardiomyocytes [100]. CAR-deficient mice are resistant to both cardiac infection and inflammation, clearly suggesting that in the acute phase of myocarditis, most of the damage is mediated by the virus. Lindner et al. demonstrated that when cardiomyocytes and cardiac fibroblasts were both infected with CVB3, cardiac fibroblasts displayed a tenfold increase in viral replication, indicating their crucial role in contributing to the viral load in myocarditis [101••].

TLR3 is involved in viral recognition and in mounting antiviral type II interferon response; mice lacking TLR3 developed severe viral myocarditis highlighting the protective action of this TLR in CVB3 infection [102]. After entering the cardiomyocytes, the viral machinery is actively replicated. Viral proteases such as enteroviral protease-2A cleave dystrophin and dystrophin-associated glycoproteins [103]. This could result in the loss of tethering of the cardiomyocytes to the ECM, leading to cardiomyocyte-ECM uncoupling [104]. Subsequent cardiomyocyte loss occurs via necrosis or apoptosis and is usually followed by replacement fibrosis. The viral PAMPs and released cellular contents are also recognized by other TLRs, and this leads to activation of other pro-inflammatory cascades [100]. The role of inflammation-induced damage in the acute phase is demonstrated by the fact that TLR4-deficient mice were protected against CVB-induced cardiac injury [105, 106••].

#### Role of the Innate and Adaptive Immune System

Infiltration of the heart by cells of the innate immune system is the hallmark of the subacute phase. Natural killer (NK) cells eliminate infected cells using cytotoxic proteins while monocytes phagocytose dead cells. Macrophages maintain their M1 phenotype in the inflammatory milieu and produce copious amounts of pro-inflammatory cytokines causing extensive tissue damage. Susceptibility to infection with CVB in animal models also appears to be sex dependent, with more severe myocarditis in males. In line with this, hearts from male animals displayed a higher number of infiltrating M1 macrophages than female hearts. The cardiac inflammatory response to infection was also enhanced when M1 macrophages, developed in vitro, were transferred into female mice. Conversely, transferring the IL10-secreting M2 macrophages, developed in vitro, into male animals inhibited cardiac disease [107]. This suggests the importance of macrophages in sex-dependent effects of CVB-induced myocardial damage.

The cells from innate immune system are eventually replaced by those from the adaptive immune system in subsequent phases, and infected cardiomyocytes are eliminated by CD8^+^ cytotoxic T cells. Severe combined immunodeficiency (SCID) animal models displayed excessive damage to cardiomyocytes by virus-mediated cardiac injury, highlighting the importance of immune cells and inflammation in elimination of viral particles [108].

Cardiac repair and remodelling follow, once the inflammatory trigger is removed. The dead tissue is replaced by a fibrotic scar facilitated by profibrotic signalling (e.g. TGFß) and the reduction in cardiac function depends on the amount of cardiomyocytes lost. However, incomplete clearance of the cardiac viral load results in chronic inflammatory activation, accelerating progression to dilated cardiomyopathy [100]. Although inflammation seems to play a crucial role in the pathophysiology of myocarditis and its sequelae, broad-scale immunosuppression fails to improve cardiac function in such patients. The other mechanism by which chronic myocardial damage can occur is through the development of autoimmune myocarditis, and IL13 seems to offer protection against experimental autoimmune myocarditis by moderating macrophage differentiation [109].

### Pressure Overload—Hypertension

Although hypertension has a strong genetic component, neurohormonal activation, oxidative stress and low-grade systemic inflammation play a vital role in its aetiology, especially in insulin-resistant states. Hypertension is a leading cause of HF and exerts a deleterious effect on the cardiovascular system through direct haemodynamic mechanisms and also through overactivation of the renin-angiotensin-aldosterone system (RAAS) [110].

Hemodynamic parameters such as increased shear stress together with low-grade systemic inflammation promotes endothelial damage in hypertension. During the course of time, this manifests itself as perivascular fibrosis with considerable deposition of collagen in the adventitia of intramural arteries, resulting in reduced vascular compliance and changes in permeability. Hypertension also elicits structural and functional changes in microcirculation leading to microvascular remodelling and rarefaction [111].

There are also simultaneous changes in the cardiac tissue; progressive deposition of collagen in cardiac ECM results in reactive interstitial fibrosis. Although this develops without cardiomyocyte loss, it decreases myocardial compliance and clinically manifests as HF with preserved ejection fraction (HFpEF) [112]. In advanced hypertension, there is a pathological hypertrophy of cardiomyocytes and also an increased loss of cardiomyocytes. This results in irreversible replacement fibrosis leading to deterioration of the systolic function of the heart, clinically manifesting as HF with reduced ejection fraction (HFrEF) [51, 110, 113]. In animal models of sudden pressure overload (e.g. TAC), the results are more dramatic with accelerated cardiomyocyte loss and more rapid onset of cardiac fibrosis [114].

RAAS is the key homeostatic hormonal mechanism that maintains blood pressure in order to ensure adequate tissue perfusion. However, stimulation of the RAAS also elicits pro-inflammatory and profibrotic responses and contributes to cardiovascular remodelling. For instance, aldosterone has been implicated in the development of cardiac fibrosis in hypertension [115]; renin overexpression in hypertensive rats leads to cardiac remodelling and diastolic dysfunction via a fibrosis-independent “titin-related” mechanism [116].

As Ang II is the key vasoconstrictive protein in this axis, we briefly discuss few novel mechanisms of Ang II-related cardiovascular remodelling. Ang II can act both independently and via the classic TGFβ axis to induce fibrosis [117]. Recent studies describe the Ang II-Gal-3-IL6 axis as a modifiable fibrotic pathway in hypertension. Genetic inhibition of IL6 resulted in reduction of cardiac inflammation and fibrosis in an Ang II high-salt-induced hypertension mouse model. IL6 deletion also improved cardiac dysfunction although there was no net reduction in blood pressure [118••], suggesting the critical role of IL6 in the mediation of cardiac inflammatory and fibrotic effects of Ang II. Subsequent studies in models of chronic Ang II-induced hypertension demonstrated that genetic ablation of Gal-3 also reduced myocardial macrophage infiltration and fibrosis, highlighting the causative role of Gal-3 in cardiac fibrosis related to hypertension [119]. ECM proteins such as osteopontin are also involved in Ang II-induced cardiac fibrosis, and studies with osteopontin^−/−^ mice indicate that there is a significant reduction in cardiac fibrosis after 3 weeks of Ang II infusion [120]. Other experimental studies, also with murine models, suggest that syndecan-1 amplifies profibrotic effects of Ang II and is a critical regulator of fibrosis in the heart [82]. Recently, neutrophil-generated S100a8/S100a9 proteins have been implicated in Ang II-induced cardiac inflammation and fibrosis [121]. There is also accumulating evidence on the role of cardiac mast cell-IL4 axis in the mediation and development of hypertension-related cardiac fibrosis [63]. Targeting these novel pathways of inflammation and fibrosis could effectively prevent or reduce cardiovascular fibrosis in the setting of hypertension.

### Heart Failure

Although most patients survive the primary cardiac event due to early detection and timely management, every cardiac insult decreases the cardiac contractile reserve and these patients have an increased risk of developing HF [122]. HF can be defined as the inability of the heart to adequately maintain cellular perfusion under normal cardiac filling pressure. While half of the patients with HF exhibit decreased ejection fraction (HFrEF), the other half have a normal EF (HFpEF). Based on clinical presentation, HF can be classified as acute HF (AHF), when the patient presents with cardiac decompensation, and CHF, when the patient has impaired cardiac function but is compensated and stable, i.e. able to maintain tissue perfusion without assistance [123, 124].

AHF is characterized by a systemic inflammatory response, with elevated pro-inflammatory cytokines [125]. Other triggers may be present that provoke inflammation: AHF is often accompanied by viral or bacterial infection and is usually preceded by MI or atrial fibrillation (AF). However, after the acute event has been treated, a chronic response develops, and such patients frequently develop CHF. Other concomitant factors such as hypertension can also contribute to the development of AHF and CHF. It is also important to note that CHF can itself be a predisposing factor for the development of future AHF, and the goal of CHF management is to maintain the patient in compensated HF state and prevent them from deteriorating into a state of acute decompensated HF (ADHF) [124].

Inflammation in the setting of CHF can be very complex. Low-grade systemic inflammation can both be a cause and consequence of HF [34, 126–128]. Moreover, chronic oxidative stress associated with HF can exacerbate the pre-existing inflammatory state [129]. It is hypothesised that the presence of co-morbidities might lead to increased inflammation and to HF [130•]. There is also upregulation of TLR4 in cardiomyocytes during HF, suggesting the direct role of cardiomyocytes in cardiac inflammation associated with HF [47••]. Sustained activation of protective neurohormonal mechanisms can also contribute to ongoing cardiac inflammation and fibrosis [131, 132••], resulting in further loss of cardiac function and clinical deterioration up to the point of ADHF and death.

**Fig. 3:**
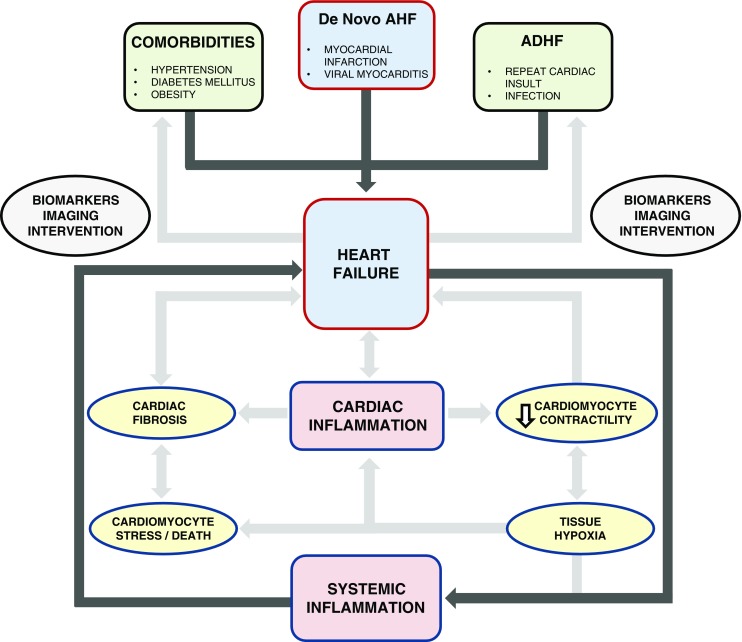
The interplay between systemic inflammation, cardiac inflammation and heart failure (HF) is highlighted. HF can arise de novo, for instance after myocardial infarction (MI) or can result from exacerbation of pre-existing HF. Long-standing systemic diseases such as hypertension, diabetes mellitus (DM) or obesity can also adversely affect cardiac function through various mechanisms (*outside the rectangular box*). HF is a systemic inflammatory state and promotes cardiac inflammation. Inflammation can affect cardiac function through several mechanisms such as (A) reduced contractility affecting mechanical properties of the heart, (B) cardiac stress leading up to cardiomyocyte death and (C) cardiac fibrosis. All these effects lead to HF or exacerbate pre-existing HF, and this is illustrated within the rectangular framework. Biomarkers and imaging can aid us in identifying the HF process early in the disease course and in assessing the nature of HF to choose appropriate therapeutic interventions. AHF, acute heart failure; ADHF, acute decompensated heart failure

Thus, it is highly relevant to address this question in patients with CHF: Is cardiac fibrosis progressive? Serial biomarker measurements and imaging modalities such as cardiac magnetic resonance imaging (CMR) can help us answer this question by aiding in identifying patients with progressive cardiac fibrosis [133–135] (Fig. 3).

## Diagnosing and Monitoring Inflammation and Fibrosis

Circulating biomarkers are biological markers detected in blood or urine that ideally reflect biochemical and (patho)physiological processes occurring in (injured) organs; they are used as an adjunct in diagnosis, prognosis and risk stratification, and also in optimizing treatment guidance [135, 136•]. Herein, we discuss the utility of biomarkers and imaging techniques in monitoring cardiac inflammation and fibrosis.

Inflammatory markers such as C-reactive protein (CRP) and IL-6 can be used to predict cardiovascular diseases and severity of HF while fibrotic markers such as Gal-3 and syndecan-1 are currently used for risk stratification in HF [137], predicting mortality [138] or readmission [139]. Serial biomarker measurements, after taking biological variability into account, could aid in monitoring the “temporal dynamics” of cardiac pathophysiological processes, thereby offering additive prognostic value [134, 135].

Utilizing circulating biomarkers to predict ongoing myocardial fibrosis could be difficult as circulating levels might not reflect ECM deposition specifically in the cardiac tissue. Their correlation with the gold standard “endomyocardial biopsy” is therefore necessary to validate them as a biomarker of myocardial fibrosis; out of a wide array of cardiac fibrosis markers, procollagen 3N-terminal peptide (P3NP) and carboxy-terminal propeptide of type 1 procollagen (P1CP) appear to have a strong correlation with histologically proven myocardial fibrosis. Lately, CMR has become the gold standard in the evaluation of cardiac fibrosis, and fibrotic biomarkers are now compared with T1-weighted contrast-enhanced CMR images [140].

Imaging can itself serve as a biomarker of cardiac inflammation and fibrosis. For instance, a T2-weighted image allows detection of oedema and cardiac inflammation during acute phases of myocarditis [141]. T1-weighted images with delayed contrast enhancement (DCE) using gadolinium can be employed to visualize inflammatory infiltrate and regional fibrosis, e.g. after MI [142]. However, such techniques lose their discriminatory power to detect diffuse interstitial fibrosis, e.g. in diabetic or hypertensive cardiomyopathy, and CMR T1 mapping is the preferred modality in such scenarios [143].

Information about cellular, molecular and metabolic events occurring in the heart can be obtained with a functional positron emission tomography (PET) scan. PET can be used to monitor myocardial metabolism using radiotracers such as ^18^F-fluorodeoxyglucose (FDG) and also to image and measure myocardial perfusion and blood flow using various PET-myocardial perfusion imaging (PET-MPI) tracers. Visualization of activated macrophages in biologically active atherosclerotic plaques or in other scenarios is also possible using this imaging technique [144••, 145]. Furthermore, PET can detect diffuse fibrosis; for example, it can be used to calculate the fraction of myocardium perfusable by water, termed perfusable tissue index (PTI). Fibrotic myocardium is unable to exchange water rapidly; hence, a decline in PTI correlates directly with the amount of fibrosis. A combination of myocardial metabolism and perfusion could also identify myocardial fibrosis more precisely. Finally, PET technology has also been harnessed to develop new HF drugs [144••].

## Therapeutic Options

Pathological cardiac remodelling can be targeted in several ways [146] but most strategies do not, or do not specifically target inflammation and fibrosis. When targeting cardiac inflammation or fibrosis, the timing of therapy will be crucial. For instance, reduction of myocardial inflammation in the initial phases of MI or during the early phases of ischaemia-reperfusion injury could potentially yield better outcomes [91•]. However, premature attenuation of fibrosis, e.g. during the onset of the proliferative phase, could result in cardiac rupture or aneurysm formation [147, 148•].

In myocarditis, chronic inflammation has been held responsible for long-term effects leading to dilatation, and cardiac macrophages have been implicated in the aetiology [107]. However, broad-scale immunosuppression fails to improve cardiac function in such patients [149]; utilizing compounds that enhance resolution might counter the chronic inflammation in such cases [17]. Addressing autoimmune mechanisms could be yet another approach to curb the progression of subclinical disease to overt dilated cardiomyopathy.

In hypertension, in addition to the existing therapy, targeting the Ang II-Gal-3-IL6 axis or the mast cell-IL4 axis using Gal-3 inhibitors or IL4 inhibitors could specifically reduce cardiac fibrosis [63, 118••, 119]. Therapeutic interventions that focus on the quality of collagen in HF could also significantly increase cardiac compliance. Excessively cross-linked collagen is difficult to degrade and critically affects ECM turnover. Syndecan-4-osteopontin-LOX axis is important in the formation of insoluble cross-linked collagen [31, 54••], and therapeutic strategies that target such pathways could also ameliorate the effects of myocardial fibrosis.

Further strategies to modulate ECM deposition are also currently being developed [146]. Modalities enhancing titin-compliance [150] and therapeutic angiogenesis [65, 67, 68] could also be employed alongside improving cardiac function in HF. This field is rapidly evolving and in the coming decade it is expected that several new drugs will enter the clinical arena.

## Conclusions

Cardiac inflammation and fibrosis are major pathophysiological mechanisms operating in the failing heart irrespective of the aetiology of HF. There is a dynamic interplay between inflammation and fibrosis in various precursors of HF such as MI, myocarditis and hypertension, and also in HF itself. Early diagnosis of HF with biomarkers and imaging is warranted; while CMR is useful for evaluating the extent of injury, serial biomarker measurements indicate if inflammation and fibrosis are progressive. A progressive disease needs an aggressive management; however, existing therapies against HF are insufficient. There is an urgent need to identify novel therapeutic targets and develop advanced therapeutic strategies to combat the syndrome of HF. To this end, exact spatio-temporal description of the elements of the inflammatory and fibrotic pathways is essential, and specific drugs that target these pathways need to be evaluated.

## Abbreviations

HF: Heart Failure
AHF: Acute Heart Failure
ADHF: Acute Decompensated Heart Failure
CHF: Chronic Heart Failure
HFpEF: Heart Failure with Preserved Ejection Fraction
HFrEF: Heart Failure with Reduced Ejection Fraction
MI: Myocardial Infarction
AF: Atrial Fibrillation
RAAS: Renin Angiotensin Aldosterone System
Ang II: Angiotensin II
LOX: Lysyl Oxidase
I/R: Ischaemia-Reperfusion Injury
TAC: Transverse Aortic Constriction
CVB: Coxsackie virus group B
CAR: Coxsackievirus and Adenovirus Receptor
DAF: Delay Accelerating Factor
IFNƴ: Interferon
NK cells: Natural Killer cells
SCID: Severe Combined Immunodeficiency
IDCM: Inflammatory Dilated Cardiomyopathy
ROS: Reactive Oxygen Species
LPS: Lipopolysaccharide
PAMP: Pathogen-Associated Molecular Pattern
DAMP: Danger-Associated Molecular Pattern
HSP: Heat-Shock Protein
TLR: Toll-like Receptor
PRR: Pathogen Recognition Receptor
PI3K: Phosphoinositide 3-kinase
IRAK-M: Interleukin-1 Receptor-Associated Kinase-M
SOCS-1: Supressor of Cytokine Signalling-1
MyD88: Myeloid Differentiation primary response gene-88
NFκβ: Nuclear Factor-κβ
RIG-1: Retinoic Acid-Inducible Gene-1
α-SMA: α-Smooth Muscle Actin
ECM: Extracellular Matrix
HA: Hyaluronic Acid
MMP: Matrix Metalloproteinase
TIMP: Tissue Inhibitor of Metalloproteinase
TGFβ: Transforming Growth Factor-β
Gal-3: Galectin 3
GAGBP: Glycosaminoglycan-Binding Protein
P3NP: Amino-terminal type III propeptide peptide
PICP: Carboxy-terminal propeptide of type I collagen
SIRT: Sirtuin
TSP: Thrombospondin
OPN: Osteopontin
SLRP: Small Leucine-Rich Proteoglycans
CRP: C-reactive Protein
PAI-1: Plasminogen Activator Inhibitor 1
uPA: Urokinase-type Plasminogen Activator
IL: Interleukin
LIF: Lukaemia Inhibitory Factor
TNFα: Tumour Necrosis Factor-α
MCP-1: Monocyte Chemoattractant Protein-1
MIP-1α: Macrophage Inflammatory Protein-1α
CMR: Cardiac Magnetic Resonance
DCE: Delayed Contrast Enhancement
PET: Positron Emission Tomography
^18^F-FDG: Fluorine-18-Fluorodeoxyglucose
PTI: Perfusable Tissue Index
